# New insights into the DNA extraction and PCR amplification of minute ascomycetes in the genus *Laboulbenia* (*Pezizomycotina*, *Laboulbeniales*)

**DOI:** 10.1186/s43008-024-00146-9

**Published:** 2024-06-11

**Authors:** Warre Van Caenegem, Danny Haelewaters

**Affiliations:** 1 Research Group Mycology, Department of Biology, Ghent University, Ghent, 9000 Belgium; 2 Meise Botanic Garden, Meise, 1860 Belgium; 3 Faculty of Science, University of South Bohemia, České Budějovice, 370 05 Czech Republic

**Keywords:** Barcoding, DNA extraction, *Laboulbenia*, *Laboulbeniales*, PCR amplification

## Abstract

Molecular studies of fungi within the order *Laboulbeniales* (*Ascomycota*, *Pezizomycotina*) have been hampered for years because of their minute size, inability to grow in axenic culture, and lack of reliable and cost-efficient DNA extraction protocols. In particular, the genus *Laboulbenia* is notorious for low success with DNA extraction and polymerase chain reaction (PCR) amplification. This is attributed to the presence of melanin, a molecule known to inhibit PCR, in the cells. We evaluated the efficacy of a standard single cell-based DNA extraction protocol by halving the recommended amount of reagents to reduce the cost per extraction and adding bovine serum albumin (BSA) during the multiple displacement amplification step to reverse the effect of melanin. A total of 196 extractions were made, 111 of which were successful. We found that halving the reagents used in the single cell-based extraction kit did not significantly affect the probability of successful DNA extraction. Using the halved protocol reduces cost and resource consumption. Moreover, there was no significant difference in the probability of successfully extracting DNA based on whether BSA was added or not, suggesting that the amount of melanin present in cells of the thallus has no major inhibitory effect on PCR. We generated 277 sequences from five loci, but amplification of the internal transcribed spacer region, the mitochondrial small subunit rDNA, and protein-coding genes remains challenging. The probability of successfully extracting DNA from Laboulbeniales was also impacted by specimen storage methods, with material preserved in > 95% ethanol yielding higher success rates compared to material stored in 70% ethanol and dried material. We emphasize the importance of proper preservation of material and propose the design of Laboulbeniales-specific primers to overcome the problems of primer mismatches and contaminants. Our new insights apply not only to the genus *Laboulbenia*; *Laboulbeniales* generally are understudied, and the vast majority of species remain unsequenced. New and approachable molecular developments will benefit the study of *Laboulbeniales*, helping to elucidate the true diversity and evolutionary relationships of these peculiar microfungi.

## Introduction

Traditionally, fungal species identification and delimitation relied on morphological characteristics, but phenotypic plasticity within species and cryptic species posed challenges (Bridge et al. 2005; Cao et al. 2021; Maharachchikumbura et al. 2021). Some groups used alternative traits like enzymatic activity or mating compatibility (Perkins and Raju 1986; Pincus et al. 2007), but these methods are not applicable to unculturable species, thus other unambiguous characters should be used. Early molecular methods based on GC-content and DNA hybridization had limited use, except in yeast studies (Bridge et al. 2005). The introduction of PCR enabled the discrimination of closely related taxa based on molecular data, revolutionizing molecular systematics in mycology (White et al. 1990; Bridge et al. 1998, 2005; Cao et al. 2021; Maharachchikumbura et al. 2021). The internal transcribed spacer of the nuclear ribosomal DNA (ITS) was proposed as the universal fungal DNA barcode marker (Schoch et al. 2012). While the ITS is nowadays often used to delimit and identify fungi, the interspecific variation in this region is too low in some groups, necessitating the use of additional markers (Stielow et al. 2015).

A particularly understudied group of fungi is the order *Laboulbeniales* (*Ascomycota*, *Pezizomycotina*). These microfungi have an obligate association with arthropod hosts. Instead of producing hyphae, *Laboulbeniales* develop a 3-D structure called a thallus from a two-celled ascospore, which attaches externally to an arthropod’s integument (Blackwell et al. 2020). Molecular studies of *Laboulbeniales* have been challenging due to the minute size of these fungi (200–300 μm on average), their melanized cells, and their inability to grow in axenic culture (Haelewaters et al. 2015; Sundberg et al. 2018a). Initial attempts using a dry ice protocol by Weir and Blackwell (2001) only had a 25% success rate. Updated versions of the same protocol were used in a few studies (Goldmann and Weir 2012, 2018; Goldmann et al. 2013). However, these protocols are time-consuming, require more than one thallus per extraction, and result in only limited success of extraction and PCR amplification (Sundberg et al. 2018a). Haelewaters et al. (2015) evaluated four DNA extraction protocols and different pre-treatments with mixed success. They had difficulties extracting DNA of *Laboulbenia* species, with success rates between 0 and 20%. DNA extraction and amplification of *Laboulbenia* species has been suggested to be particularly difficult, because many species in the genus have melanized cells, which is known to interfere with DNA polymerase during PCR (Eckhart et al. 2000; Gibson 2012; Haelewaters et al. 2015; Sundberg et al. 2018a). Several PCR inhibitor removal kits are available to remove molecules such as melanin and phenols (Hu et al. 2015; Vicente et al. 2019). Similar results can be reached by adding bovine serum albumin (BSA) to the PCR mixture (Giambernardi et al. 1998), but this has not yet been tested for *Laboulbenia* species nor *Laboulbeniales* in general.

Sundberg et al. (2018a) developed a new DNA extraction protocol, in which a manual press system was used to disrupt the tough cell walls of *Laboulbeniales*. Using one thallus per DNA extraction, they successfully obtained 156 sequences: 20 nrSSU, 56 ITS, 59 nrLSU, and 21 mtSSU. Their protocol does not involve any other treatment of the thalli, which is a major advantage compared to the other described methods. However, drawbacks for their protocol include the need for custom-made components and the fact that DNA extractions are fully depleted during PCR amplification and thus cannot be stored.

Haelewaters et al. (2018a) published another method to extract DNA of *Laboulbeniales*. Using the REPLI-g Single Cell Kit (Qiagen), a sufficient amount of DNA can be obtained from a single thallus. This protocol is different from the previous methods, as it involves a whole-genome amplification (WGA) step. Due to the WGA, there is a higher risk to amplify contaminants. Yet, it has been successfully used in many other studies by Haelewaters and colleagues (Haelewaters et al. 2018b, 2019a, 2019b, 2022; Walker et al. 2018; Haelewaters and Pfister 2019; Haelewaters and De Kesel 2020; Liu et al. 2020; Van Caenegem et al. 2023a, 2023b). Haelewaters et al. (2019b) used a modified protocol, in which they halved the use of every component, to save products and reduce costs per extraction. There are doubts about the effectiveness of this modified protocol (D. Haelewaters and P. Mironova, pers. comm.), but no formal tests have been performed to evaluate the significance of these doubts.

Currently, the nuclear small and large subunit ribosomal RNA (nrSSU and nrLSU), the internal transcribed spacer region (ITS), the minichromosome maintenance complex component 7 protein-coding gene (*MCM7*), the translation elongation factor 1α protein-coding gene (*TEF1*), and the mitochondrial small subunit rRNA (mtSSU) have been sequenced for several species of *Laboulbeniales* (Goldmann and Weir 2012; Goldmann et al. 2013; Haelewaters et al. 2015, 2018a, 2019b, 2022; Sundberg et al. 2018a, 2018b; Liu et al. 2020; Van Caenegem et al. 2023a, 2023b). General fungal primers designed by White et al. (1990) have mainly been used to amplify regions of ribosomal RNA (nrSSU, ITS, nrLSU). Haelewaters et al. (2015) developed a *Laboulbeniomycetes*-specific nrSSU primer pair. Additionally, recent research reported low amplification of the ITS region using general fungal primers, which resulted in the design of a *Hesperomyces*-specific ITS primer pair (ITShespL and ITShespR) and the *Laboulbeniomycetes*-specific LabITS1 forward primer (Haelewaters et al. 2018a, 2019b).

Only 10 nrSSU sequences of 9 species, 12 ITS sequences of 4 species, and 34 nrLSU sequences of 12 species of *Laboulbenia* are available in GenBank. Given that almost 700 species are described (Haelewaters et al. 2024), there is a huge discrepancy between described and sequenced species of *Laboulbenia*. The main goal of this study was to generate DNA sequences of *Laboulbenia* species for future molecular studies, given their paucity in public sequence databases. We had the opportunity to test questions regarding DNA extraction protocols, primer pairs, and PCR protocols. During the quest to consistently extract and sequence DNA of *Laboulbenia*, we (1) researched how the preservation methods correlate with DNA extraction success, (2) explored the boundaries of the REPLI-g Single Cell Kit by halving the amount of reagents per extraction, (3) tested whether the addition of BSA results in more successful DNA extractions, and (4) identified the usefulness of different primer pairs and PCR protocols for multiple loci to successfully generate high-quality DNA sequences of *Laboulbenia* species.

## Methods

### Collection and processing of beetles

Beetles (*Coleoptera*) were collected using different entomological methods (light traps, pitfall traps, mouth-operated aspirator, and hand collection) by the authors and by entomologists who sent specimens for study of their *Laboulbeniales*. Specimens were collected in 70% to 99% ethanol. All specimens were transferred to 99% ethanol upon arrival in the lab at Ghent University. Beetles were screened for the presence of *Laboulbeniales* using an RZB-PL 65.500 stereoscope (Novex, Arnhem, The Netherlands). Infected specimens were identified by the authors (using Muilwijk et al. 2015) or their collectors. Other host specimens were sent to Dr. Menno Schilthuizen (Taxon Expeditions, Leiden, The Netherlands) for identification and subsequent vouchering. Specimens are stored in the collection of Taxon Expeditions (TXEX, Leiden, The Netherlands) or the entomology collections of the Royal Belgian Institute of Natural Sciences (KBIN, Brussels, Belgium).

### Morphological study of *Laboulbeniales*

*Laboulbeniales* microfungi were mounted in permanent slides as described by Liu et al. (2020). A 1:1 mixture of Hoyer’s medium and glycerin was used, as pure Hoyer’s medium dries too quickly. A small droplet of water was placed on a microscope slide, on which a 22 × 22 mm coverslip was put. The purpose of this was to ensure that the 22 × 22 mm coverslip was somewhat stuck to the microscope slide during further manipulation and thus could not move unexpectedly or fall. A droplet of the Hoyer’s/glycerin mixture was placed off-center on the coverslip. A hypodermic needle was used to remove *Laboulbeniales* thalli from the host and place them in the droplet. Thalli were arranged in one row or multiple rows in the middle of the coverslip. A smaller 18 × 18 mm coverslip with a drop of Amann’s medium was flipped upside down (drop of Amann’s medium facing down) and positioned over the thalli by gently lowering it with a dissecting pin. Next, the corners of the 18 × 18 mm coverslip were sealed to the larger coverslip with nail polish. Solakryl BMX (Ento Sphinx, Pardubice, Czech Republic) was applied to the microscope slide, and the coverslip assembly with the smaller coverslip facing downwards was slowly lowered and gently placed sideways on the microscope slide. Our permanent slides are each composed of a 22 × 22 mm coverslip on top of an 18 × 18 mm coverslip, with the thalli in between those two coverslips, and the microscope slide at the bottom.

Mounted thalli were viewed at 100–400 × magnification under an Olympus BH-2 microscope (Olympus, Center Valley, PA). Thalli were identified based on relevant literature (Thaxter 1896; Majewski 1994; De Kesel et al. 2020; Haelewaters and De Kesel 2020; Santamaria and Pedersen 2021) and supplementary papers (Santamaria et al. 1991). Permanent slides of *Laboulbeniales* are deposited in the Herbarium Universitatis Gandavensis (GENT).

### DNA extraction, PCR amplification, and sequencing

**Fig. 1:**
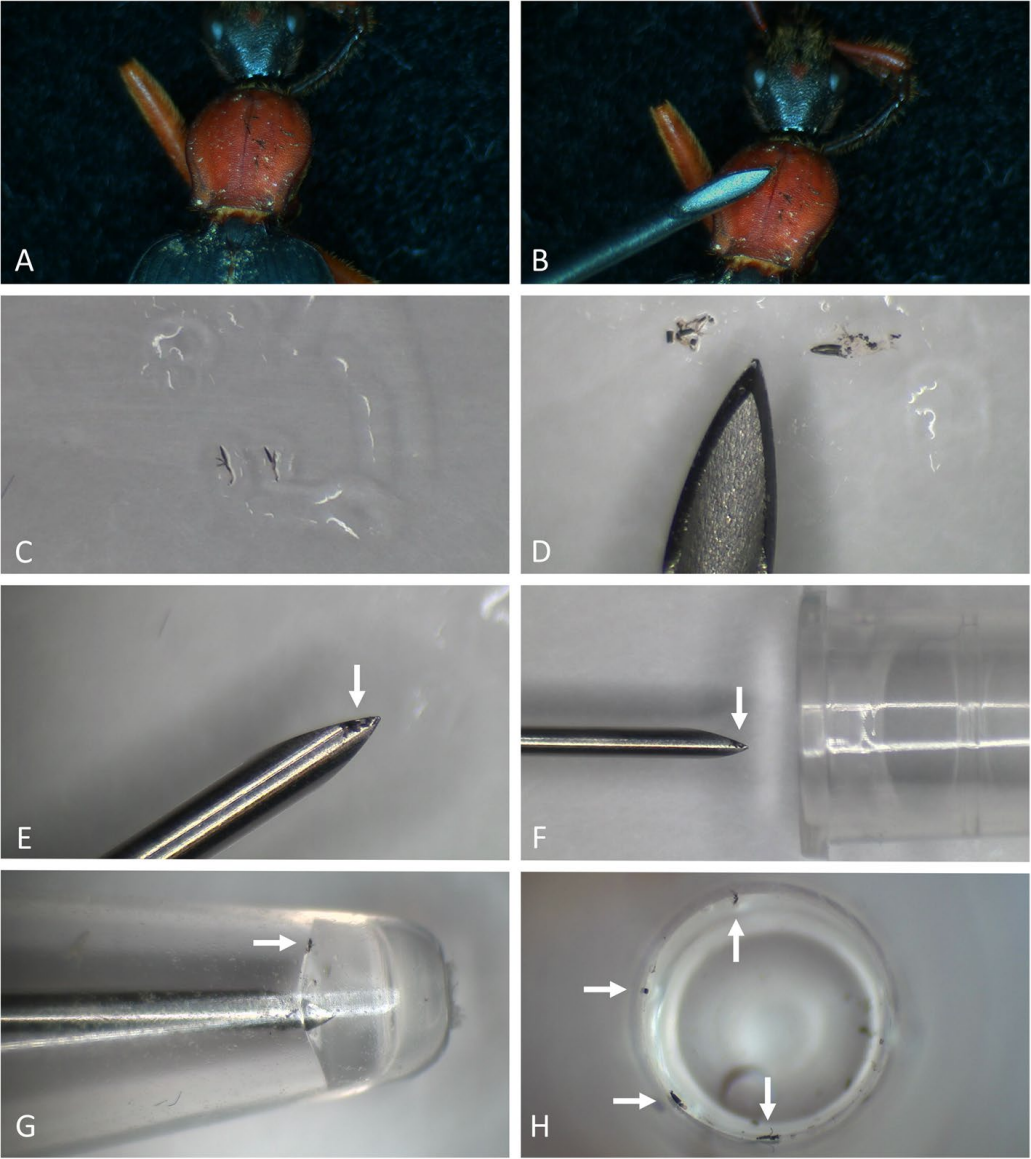
Illustration of the initial steps to perform DNA extractions using the REPLI-g Single Cell kit (Qiagen). **A** Thalli of *Laboulbenia galeritae* attached to the pronotum of *Galerita bicolor*. **B** The thalli are removed from their host using a hypodermic needle.** C** The removed thalli are placed in a droplet of glycerin. **D** Thalli are cut in multiple pieces using a surgical blade (left) or crushed (right) using the sharp tip of a hypodermic needle. **E** The pieces are gathered onto the tip of the needle. **F** The needle is carefully moved inside the PCR tube, while keeping a clear vision through the dissecting microscope. Make sure that there is still a bit of glycerin mixed with the pieces of thalli. This will prevent the loss of thalli due to static electricity during transfer. **G** The tip of the needle is inserted into the PBS buffer. This will result in the pieces of thalli releasing from the tip almost immediately. Eventually, stirring can help to make sure the pieces come off the tip of the needle. **H** It is recommended to visually check if the pieces of thalli are present in the PCR tube. Arrows indicate the pieces of thalli of *L. galeritae*

DNA extractions were done using the REPLI-g Single Cell Kit (Qiagen, Stanford, CA). The initial steps are described in Fig. 1. Molecular work was done at the Centre for Molecular Phylogeny and Evolution (CeMoFE) at the Ledeganck Campus of Ghent University. All steps were performed wearing disposable latex gloves. To avoid contamination, hypodermic needles for micromanipulation of thalli were thoroughly cleaned with 70% ethanol and bleach before and after every prepared extraction. Thalli of *Laboulbeniales* were removed from their host under a dissecting microscope, using a needle inserted onto a glass syringe for holdfast. The tip of the needle was submerged in glycerin to prevent thalli from flying away during transfer. The removed thalli were placed in a droplet of glycerin on a microscope slide. Appendages were often cut off to avoid downstream contamination, as they may harbor debris including fungal propagules. The thalli were either cut into multiple smaller pieces using the sharp tip of the hypodermic needle (sensu Haelewaters et al. 2018a), crushed by pressing the tip of the hypodermic needle onto the thalli (sensu Weir and Blackwell 2001; Sundberg et al. 2018a), or a combination of both techniques was used. These pieces were then placed in a 0.2 ml PCR tube with 4 µl of phosphate-buffered saline (PBS). Next, 3 µl of prepared D2 buffer was pipetted against the inner wall of the PCR tube to prevent accidental removal of thallus fragments, and the tube was shortly centrifuged. The tube was then incubated at 65 °C for 30 min. After incubation, the tube was centrifuged for 20 min at 4000 RPM, and 3 µl of STOP solution was added. Again, to prevent accidental removal of thallus fragments, the STOP solution was pipetted against the inner wall of the PCR tube, followed by a brief centrifugation step. From here on, two different protocols were followed:

- 1. The normal REPLI-g protocol: in the tube, 29 µl Reaction Buffer; 9 µl ddH_2_O; and 2 µl REPLI-g sc DNA Polymerase was added, as indicated in the manufacturer’s instructions (Qiagen).
- 2. The halved REPLI-g protocol: in the tube, 14.5 µl Reaction Buffer; 4.5 µl ddH_2_O; and 1 µl REPLI-g sc DNA Polymerase was added.

We also made 32 extractions that we supplied with 21.75 µl Reaction Buffer; 6.75 µl ddH_2_O; and 1.5 µl REPLI-g sc DNA Polymerase (a total of 30 µl). Initially, we wanted to test whether the probability to successfully extract DNA differed significantly between these three protocols (normal protocol, halved protocol, and 30 µl protocol). After the first tests, we already observed that this probability did not differ significantly, and we decided to simplify and streamline our experimental setup by focusing on the two extremes (comparing the normal and the halve protocol).

To test the effect of BSA during the whole genome amplification step of the REPLI-g Single Cell Kit, we arbitrarily added 5 µl BSA (20 mg/ml, stock concentration).

After the addition of all reagents, the samples were incubated at 30 °C for 8 h. During this incubation step, whole-genome amplification (WGA) took place: the whole genome DNA in the tubes was amplified using Multiple Displacement Amplification (MDA) (see Discussion). After the WGA, the polymerase was inactivated at 65 °C for 3 min. DNA extractions were stored at -20 °C. DNA quantification was done using the Qubit 2.0 fluorescence spectrometer (Thermo Fisher Scientific, Waltham, MA) and measurements of the A260/A280 and A260/230 absorbance ratios were taken using a NanoDrop 2000 (Thermo Fisher Scientific).

The nrSSU, ITS, and nrLSU were amplified. Additionally, we attempted to amplify *MCM7*, *TEF1*, and mtSSU. All primer pairs used are given in Table 1. PCR reactions (25 µl total) consisted of 13.3 µl of RedExtract *Taq* polymerase (Sigma-Aldrich), 2.5 µl of each 10 µM primer, 5.45 µl of ddH_2_O, and 1 µl of DNA extract. Before pipetting 1 µl of DNA extract, the PCR tube was vortexed briefly. PCR conditions are listed in Table 2. PCR products were stored at -20 °C. We also attempted to amplify additional nrLSU, ITS, and *TEF1* sequences of older, preserved extractions, which were made during former studies (Haelewaters 2018; De Weggheleire 2019; Haelewaters et al. 2019a).

**Table 1:** Primer pairs used in this study, including their PCR products and references

Forward primer	Reverse primer	PCR product	Reference forward primer	Reference reverse primer
NSL1	NSL2	nrSSU	Haelewaters et al. (2015)	Haelewaters et al. (2015)
SL122	NSL2	nrSSU	Landvik et al. (1997)	Haelewaters et al. (2015)
ITS1f	ITS4	ITS	Gardes and Bruns (1993)	White et al. (1990)
ITS5	ITS4	ITS	White et al. (1990)	White et al. (1990)
ITS3	ITS4	ITS2	White et al. (1990)	White et al. (1990)
LR0R	LR5	nrLSU	Hopple (1994)	Vilgalys and Hester (1990)
NL1	NL4	nrLSU	Kurtzman and Robnett (1997)	Kurtzman and Robnett (1997)
LIC24R	LR3	nrLSU	Miadlikowska and Lutzoni (2000)	Vilgalys and Hester (1990)
MCM7-709for	MCM7-1384rev	*MCM7*	Schmitt et al. (2009)	Schmitt et al. (2009)
EF1-1018F (al33f)	EF1-1620R	*TEF1*	Stielow et al. (2015)	Stielow et al. (2015)
al33_alternative_f	EF1-1620R	*TEF1*	Stielow et al. (2015)	Stielow et al. (2015)
MS1	MS2	mtSSU	White et al. (1990)	White et al. (1990)

**Table 2:** PCR conditions for each targeted locus

**nrSSU**	**ITS**	**nrLSU**	**CombSIL(** **Combination** **SSU,** **ITS, and** **LSU)**
94 °C for 5 min 39 cycles of 94 °C for 30 s 50 °C for 45 s 72 °C for 1:30 min 72 °C for 10 min	94 °C for 3 min 34 cycles of 94 °C for 1 min 50 °C for 45 s 72 °C for 1:30 min 72 °C for 10 min	94 °C for 5 min 34 cycles of 94 °C for 30 s 50 °C for 45 s 72 °C for 1 min 72 °C for 7 min	94 °C for 5 min 39 cycles of 94 °C for 1 min 50 °C for 45 s 72 °C for 1:30 min 72 °C for 10 min
***MCM7*** 94 °C for 5 min 10 cycles of 94 °C for 45 s 55 °C (-1 °C/cycle) for 50 s 72 °C for 1 min 24 cycles of 94 °C for 45 s 47 °C for 50 s 72 °C for 1 min 72 °C for 5 min	***TEF1*** 94 °C for 5 min 10 cycles of 94 °C for 50 s 54 °C (-1 °C/cycle) for 50 s 72 °C for 50 s 40 cycles of 94 °C for 50 s 48 °C for 50 s 72 °C for 50 s 72 °C for 7 min	***TEF1* New** 94 °C for 5 min 10 cycles of 94 °C for 50 s 54 °C (-1 °C/cycle) for 50 s 72 °C for 1 min 40 cycles of 94 °C for 50 s 53 °C for 50 s 72 °C for 1 min 72 °C for 7 min	**mtSSU** 94 °C for 5 min 38 cycles of 94 °C for 30 s 48–65 °C for 45 s 72 °C for 1:30 min 72 °C for 7 min

Following PCR, gel electrophoresis was performed by loading the PCR products on a Tris–acetate-EDTA (TAE) 1% agarose gel at 130 V for 30 min. The gels were placed in an ethidium bromide solution for 15 min to visualize the PCR products. Purification of PCR products was done using 1.5 µl of Exo-FAP (0.5 µl Exonuclease I, 1 µl FAST Alkaline Phosphatase) (Thermo Fisher Scientific) per 10 µl of PCR product, at 37 °C for 15 min, followed by deactivation at 85 °C for 15 min. Purified PCR products were sequenced using an automated ABI 3730XL capillary sequencer at Macrogen (Amsterdam, The Netherlands). Sequencing primers were the same as the primers used to amplify the region of interest. Forward and reverse sequence reads were assembled and edited in Sequencher version 5.4.6 (Gene Codes Corporation, Ann Arbor, MI). Newly generated sequences were submitted to NCBI GenBank.

To assess the identity of the newly generated sequences, we constructed four alignments (nrSSU, ITS nrLSU, and TEF1) of these sequences, supplemented with a broad range of *Laboulbeniomycetes* sequences available on NCBI Genbank. We aligned nrSSU, nrLSU, and *TEF1* sequences by locus with the G-INS-i strategy and ITS sequences with the E-INS-i strategy using the online version 7 of MAFFT (Kuraku et al. 2013; Katoh et al. 2019). Models for nucleotide substitution were selected for each partition with ModelFinder (Kalyaanamoorthy et al. 2017) according to the corrected Akaike Information Criterion (AICc). Maximum likelihood (ML) was inferred using IQ-TREE (Nguyen et al. 2015) under partitioned models (Chernomor et al. 2016). Ultrafast bootstrapping was performed with 1000 replicates (Hoang et al. 2018). Alignments and resulting phylogenetic trees are available on GitHub: https://github.com/dannyhaelewaters/teamlaboul/tree/main/molecular_laboulbenia_paper.

### Statistical analyses

To test the difference in success/fail ratio between methods of preservation and whether the probability to successfully extract DNA differs between the halved and the normal REPLI-g protocol, we used generalized linear mixed models as implemented in the *lme4* package in R (Bates et al. 2015; R Core Team 2021). We tested the assumptions for these models using the *DHARMa* package (Hartig 2022). Three methods of preservations were defined: ‘*doubtful*’ (specimens that were collected and preserved in 70% ethanol or dried and pinned for a prolonged time); ‘*uncertain*’ (specimens for which the preservation method was unknown); and ‘*good*’ (specimens that were collected and preserved in > 95% ethanol). The ‘*uncertain*’ group was created to include specimens from which the preservation method was unknown, to prevent assigning a specimen to a wrong group (to ‘*doubtful*’ or ‘*good*’). To compare the means of DNA concentration between the halved and the normal REPLI-g protocol and whether the addition of BSA increases the probability to successfully extract DNA, we used linear mixed models as implemented in the *lme4* package in R (Bates et al. 2015; R Core Team 2021). Graphical representation of data was made using the *ggplot2* package (Wickham 2016).

We included the species of *Laboulbenia* as a random effect, because success of DNA extraction might be correlated with species, as each species has a different degree of melanization. It is important to note that most species were only represented once or only a few times, so there was no balanced design. We assigned an extraction as ‘*successful*’ when there was at least a clear single band of the nrSSU amplicon on the gel or when the ITS or nrLSU sequence matched with *Laboulbenia* species. We used the *emmeans* package to obtain the Estimated Marginal Means (EMM) for each group and to compare the means between groups (Lenth et al. 2024). R code, output of the analyses, and additional exploratory figures can be found on GitHub: https://github.com/dannyhaelewaters/teamlaboul/tree/main/molecular_laboulbenia_paper.

## Results

### Comparison of preservation methods

**Fig. 2:**
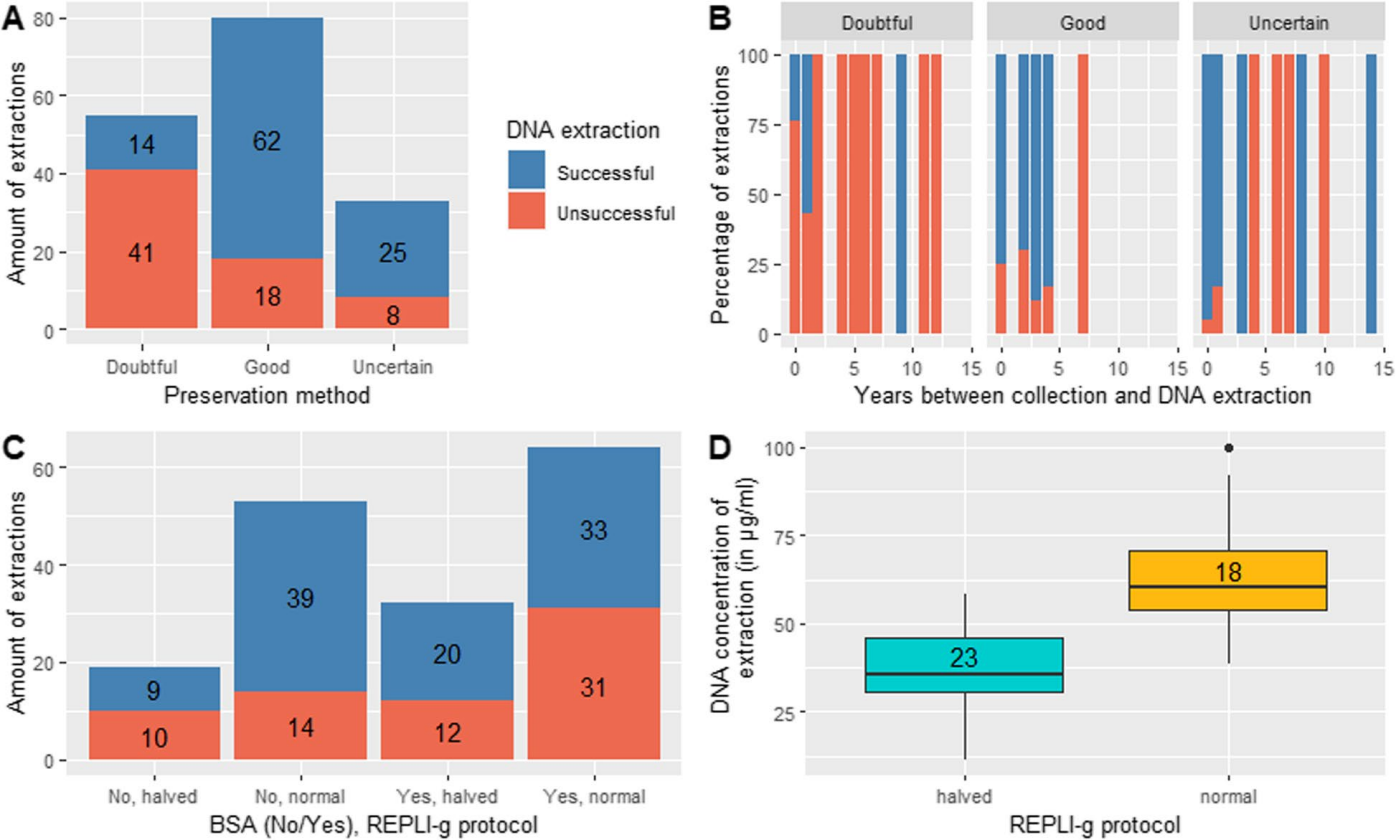
Results of comparison of different preservation techniques and protocols to improve efficacy of successfully extracting DNA from thalli of *Laboulbenia*. **A** Bar plots indicating the number of extractions in each preservation group, showing successful (in blue) and unsuccessful (in red) DNA extractions. **B** Bar plots for each preservation method separately, indicating the percentage of successful (in blue) and unsuccessful (in red) DNA extractions in relation to the period (in years) between the collection date and the date on which the DNA extraction was performed. **C** Bar plots of successful (in blue) and unsuccessful (in red) DNA extractions for each combination of BSA addition (no or yes) and REPLI-g protocol (halved or normal).** D** Box plots showing the variation in DNA concentration of extractions made with the normal and halved REPLI-g protocol, with indication of the number of samples per group

A total of 196 extractions were made, of which 111 were successful. The probability of successfully extracting DNA of thalli from ‘doubtful’ specimens was significantly lower (π = 0.167, 95% confidence interval (CI): 0.0669–0.360) compared to ‘good’ specimens (π = 0.824, 95% CI: 0.6581–0.919) (Fig. 2A). There was no obvious pattern visible between DNA extraction success and the time (both in months and years) between collection and DNA extraction (Fig. 2B).

### Comparison of extraction protocols and DNA concentration

There was no significant difference in probability of ‘*success*’ between protocols, except between the ‘*No BSA and halved*’ and ‘*BSA and halved*’ groups (Fig. 2C). The EMM of the probabilities and their CIs are given in Table 3. There was a significant difference in DNA concentration between the halved and normal REPLI-g protocols (*p* < 0.001) (Fig. 2D). The DNA concentration was significantly higher with the normal REPLI-g protocol (29 µl Reaction Buffer, 9 µl ddH_2_O, and 2 µl REPLI-g sc DNA Polymerase added, totaling 40 µl) (EMM = 63.4 µg/ml, 95% CI: 56.1–70.7) compared to the halved REPLI-g protocol (only 20 µl mixture added) (EMM = 37.2 µg/ml, 95% CI: 30.9–43.5). The A260/A280 and A260/A230 ratios of 45 DNA extractions (33 undiluted and 12 1/10 diluted) were measured. Only one of the undiluted DNA extractions had an A260/A280 ratio within the accepted range of ‘pure’ DNA (1.7–2.0). For the diluted DNA extractions, 10 extractions had an A260/A280 ratio of around 1.8, and 4 of these were also within the range of the ideal A260/A230 ratio (1.9–2.2).

**Table 3:** The Estimated Marginal Mean probability of ‘*success*’ for each DNA extraction protocol, and its 95% confidence interval (CI)

BSA added and REPLI-g protocol	Probability of ‘*success*’	95% CI
No BSA and halved	0.300	0.104–0.613
BSA and halved	0.852	0.595–0.958
No BSA and normal	0.709	0.471–0.869
BSA and normal	0.668	0.447–0.833

### Evaluation of primer pairs, PCR protocols, and sequence success

Of the 111 successful extractions, 16 were contaminated and we could only generate sequences of the nrSSU region with the *Laboulbeniales*-specific NLS1/NSL2 primers. The nrSSU sequences of these contaminated extractions were of good quality. The contaminants were identified using ITS or nrLSU sequences and are shown in Table 4. From the successful extractions, we generated 104 nrSSU, 64 ITS, 92 nrLSU, 1 *MCM7*, and 16 *TEF1* sequences (Tables 5 and 6). We also generated 1 nrSSU, 1 ITS, 4 nrLSU, and 5 *TEF1* sequences of preserved extractions from former studies (Haelewaters 2018; De Weggheleire 2019; Haelewaters et al. 2019a). All generated sequences were submitted to NCBI Genbank and their accession numbers are presented in Table 6.

**Table 4:** Contaminants in the DNA extractions, with indications of the phylum, order, family, and the number of extractions that were contaminated with these species

Species	Phylum	Order, family	Number of encounters
*Akanthomyces muscarius*	*Ascomycota*	*Hypocreales*, *Cordycipitaceae*	1
*Apiotrichum lignicola*	*Basidiomycota*	*Trichosporonales*, *Trichosporonaceae*	1
*Cladosporium tenuissimum*	*Ascomycota*	*Capnodiales*, *Cladosporiaceae*	2
*Leptospora rubella*	*Ascomycota*	*Dothideomycetes incertae sedis*	1
*Malassezia restricta*	*Basidiomycota*	*Malasseziales*, *Malasseziaceae*	1
*Malassezia sympodialis*	*Basidiomycota*	*Malasseziales*, *Malasseziaceae*	3
*Priceomyces vitoshaensis*	*Ascomycota*	*Saccharomycetales*, *Debaryomycetaceae*	5
Unidentified *Chaetothyriales* sp.	*Ascomycota*	*Chaetothyriales*	1
Unidentified *Sporidiobolaceae* sp.	*Ascomycota*	*Sporidiobolales*, *Sporidiobolaceae*	1

**Table 5:** Primer pairs, their PCR products, and the number of sequences generated during this study using each of those primer combinations

Forward primer	Reverse primer	PCR product	Number of sequences generated
NSL1	NSL2	nrSSU	97
SL122	NSL2	nrSSU	7
ITS1f	ITS4	ITS	15
ITS5	ITS4	ITS	4
ITS3	ITS4	ITS2	45
LR0R	LR5	nrLSU	16
NL1	NL4	nrLSU	69
LIC24R	LR3	nrLSU	7
MCM7-709for	MCM7-1384rev	*MCM7*	1
EF1-1018F (al33f)	EF1-1620R	*TEF1*	13
al33_alternative_f	EF1-1620R	*TEF1*	3
MS1	MS2	mtSSU	0
			277

**Table 6:** Sequences generated in this study, with an overview of the *Laboulbenia* species, its host species, country of record, and the loci with their accession numbers. Asterisks indicate isolates that were already made in former studies and for which additional sequences were generated here

Isolate	Species	Host species	Country	nrSSU	ITS	nrLSU	*MCM7*	*TEF1*
D. Haelew. 4479b	*Laboulbenia anoplogenii*	*Stenolophus mixtus* (Herbst, 1784)	Belgium	PP620867	PP626208	PP620952		
D. Haelew. 3035a	*Laboulbenia argutoris*	*Pterostichus strenuus* (Panzer, 1796)	Belgium	PP620868	PP626209	PP620953		
D. Haelew. 3768a	*Laboulbenia argutoris*	*Pterostichus strenuu*s	Belgium	PP620869				
D. Haelew. 4095a	*Laboulbenia argutoris*	*Pterostichus strenuus*	Belgium	PP620870	PP626210	PP620954		
D. Haelew. 4465b	*Laboulbenia argutoris*	*Pterostichus strenuus*	The Netherlands		PP626211	PP620955		
D. Haelew. 3758a (ADK6522)	*Laboulbenia benjaminii*	*Badister unipustulatus* Bonelli, 1813	Belgium	OR680738	OR680744	OR680759		OR762491
D. Haelew. 1229b	*Laboulbenia bicornis*	Gyrinidae sp.	Uganda	PP620871		PP620956		
D. Haelew. 4333a	*Laboulbenia bicornis*	Gyrinidae sp.	Uganda	OR680728		OR680748		
D. Haelew. 1346b*	*Laboulbenia bruchii*	*Neolema adunata* White, 1993	Panama		OR680724			
D. Haelew. 1007a*	*Laboulbenia calathi*	*Calathus melanocephalus* (Linnaeus, 1758)	The Netherlands			OR680755		
D. Haelew. 1746a	*Laboulbenia casnoniae*	*Colliuris pensylvanica* (Linnaeus, 1758)	United States of America	PP620872	PP626212	PP620957	PP601376	PP601362
D. Haelew. 4194b	*Laboulbenia* cf. *disonichae*	*Acanthonycha* sp.	Panama	PP620873		PP620958		PP601363
D. Haelew. 4194c	*Laboulbenia* cf. *dorstii*	*Acanthonycha* sp.	Panama	PP620874		PP620959		
D. Haelew. 3976a	*Laboulbenia clivinalis*	*Clivina fossor* (Linnaeus, 1758)	Belgium	PP620875	PP626213	PP620960		
D. Haelew. 3037a (ADK6493)	*Laboulbenia clivinalis*	*Clivina fossor*	Latvia	OR680736	OR680742	OR680757		
D. Haelew. 4252a	*Laboulbenia clivinalis*	*Clivina fossor*	The Netherlands	PP620876				
D. Haelew. 3038a (ADK6459)	*Laboulbenia collae*	*Paranchus albipes* (Fabricius, 1796)	Belgium	PP620877		PP620961		
D. Haelew. 3038b (ADK6459)	*Laboulbenia collae*	*Paranchus albipes*	Belgium	OR680732	OR680739	OR680752		
D. Haelew. 4101b	*Laboulbenia collae*	*Paranchus albipes*	Belgium	PP620878	PP626214	PP620962		
D. Haelew. 4308b	*Laboulbenia collae*	*Paranchus albipes*	Portugal	PP620879	PP626215	PP620963		PP601364
D. Haelew. 3759a (ADK6524)	*Laboulbenia coneglianensis*	*Harpalus griseus* (Panzer, 1796)	Belgium	OR680734	OR680741	OR680754		
D. Haelew. 3970a	*Laboulbenia cristata*	*Paederus littoralis* Gravenhorst, 1802	Belgium	OR680735		OR680756		
D. Haelew. 3970b	*Laboulbenia cristata*	*Paederus littoralis*	Belgium	PP620880		PP620964		
D. Haelew. 3970c	*Laboulbenia cristata*	*Paederus littoralis*	Belgium	PP620881				
D. Haelew. 3970d	*Laboulbenia cristata*	*Paederus littoralis*	Belgium	PP620882		PP620965		
D. Haelew. 3770a	*Laboulbenia cristata*	*Paederus riparius* (Linnaeus, 1758)	Belgium	PP620883		PP620966		
D. Haelew. 4103a	*Laboulbenia elaphricola*	*Elaphrus aureus* P. Müller, 1821	Latvia	PP620884	PP626216	PP620968		
D. Haelew. 4179a	*Laboulbenia elongata*	*Agonum extensicolle* (Say, 1823)	United States of America	PP620885	PP626217	PP620969		
D. Haelew. 4183a	*Laboulbenia elongata*	*Agonum extensicolle*	United States of America	PP626218	PP620970			
D. Haelew. 4184a	*Laboulbenia elongata*	*Agonum extensicolle*	United States of America	PP620886	PP626219	PP620971		
D. Haelew. 4187b	*Laboulbenia elongata*	*Agonum extensicolle*	United States of America	PP620887	PP626220	PP620972		
D. Haelew. 4093a	*Laboulbenia eubradycelli*	*Bradycellus verbasci* (Duftschmid, 1812)	Belgium	PP620888	PP626221	PP620973		
D. Haelew. 4196b	*Laboulbenia eubradycelli*	*Bradycellus verbasci*	Belgium	PP620889	PP626222	PP620974		PP601365
D. Haelew. 4208a	*Laboulbenia eubradycelli*	*Bradycellus verbasci*	France	PP620890				
D. Haelew. 4363a	*Laboulbenia expectata* nom. prov.	*Pterostichus vernalis* (Panzer, 1796)	Belgium	OR723991	OR752334	OR752347		
D. Haelew. 4483a	*Laboulbenia expectata* nom. prov.	*Pterostichus vernalis*	Belgium	OR723993	OR752337	OR752345		
D. Haelew. 3044a (ADK6487)	*Laboulbenia fasciculata*	*Patrobus atrorufus* (Ström, 1768)	Belgium	OR680729	OR680723	OR680749		
D. Haelew. 3045a	*Laboulbenia fasciculata* var. *omophroni*	*Omophron limbatum* (Fabricius, 1777)	Latvia	PP620891	PP626223	PP620975		PP601366
D. Haelew. 4480a	*Laboulbenia flagellata*	*Agonum emarginatum* (Gyllenhal, 1827)	Belgium	OR723995	OR752335	OR752343		
D. Haelew. 4733a	*Laboulbenia flagellata*	*Agonum fuliginosum* (Panzer, 1809)	Belgium	OR723994	OR752338	OR752346		
D. Haelew. 1457a* (ADK6337)	*Laboulbenia flagellata*	*Agonum micans* (Nicolai, 1822)	Belgium					OR762495
D. Haelew. 1457b* (ADK6337)	*Laboulbenia flagellata*	*Agonum micans*	Belgium					OR762496
D. Haelew. 3769a (ADK6535)	*Laboulbenia flagellata*	*Agonum muelleri* (Herbst, 1784)	Belgium	OR723990		OR752342		OR762492
D. Haelew. 4538a	*Laboulbenia flagellata*	*Oxypselaphus obscurus* (Herbst, 1784)	Belgium	OR723992	OR752336	OR752344		
D. Haelew. 4099a (ADK6459)	*Laboulbenia flagellata*	*Paranchus albipes*	Belgium	OR723988	OR752332	OR752340		
D. Haelew. 4101a (ADK6459)	*Laboulbenia flagellata*	*Paranchus albipes*	Belgium	OR723989	OR752333	OR752341		
D. Haelew. 1454a* (ADK6329)	*Laboulbenia flagellata*	*Platynus assimilis* (Paykull, 1790)	Belgium					OR762493
D. Haelew. 1454b* (ADK6329)	*Laboulbenia flagellata*	*Platynus assimilis*	Belgium					OR762494
D. Haelew. 3966a	*Laboulbenia flagellata*	*Platynus assimilis*	Belgium	OR723987	OR752331	OR752339		
D. Haelew. 4600a	*Laboulbenia flagellata*	*Platynus assimilis*	Belgium	PP620892				
D. Haelew. 4181a	*Laboulbenia galeritae*	*Galerita bicolor* (Drury, 1773)	United States of America	PP620893		PP620976		PP601367
D. Haelew. 4182b	*Laboulbenia galeritae*	*Galerita bicolor*	United States of America	PP620894		PP620977		PP601368
D. Haelew. 4154a	*Laboulbenia giardi*	*Dicheirotrichus gustavii* Crotch, 1871	Belgium	OR680726		OR680746		
D. Haelew. 3052a (ADK6491)	*Laboulbenia giardii*	*Dicheirotrichus gustavii*	Belgium	OR680727		OR680747		
D. Haelew. 4170a	*Laboulbenia giardii*	*Dicheirotrichus obsoletus* (Dejean, 1829)	Belgium	PP620895	PP626224	PP620978		
D. Haelew. 4489a	*Laboulbenia gyrinicola*	*Gyrinus substriatus* Stephens, 1829	The Netherlands	PP620896		PP620979		
D. Haelew. 4489b	*Laboulbenia gyrinicola*	*Gyrinus substriatus*	The Netherlands	PP620897		PP620980		
D. Haelew. 4490a	*Laboulbenia gyrinicola*	*Gyrinus substriatus*	The Netherlands	PP620898				
D. Haelew. 3755a	*Laboulbenia hyalopoda*	*Paradromius linearis* (Olivier, 1795)	Belgium	PP620899	PP626225	PP620981		PP601369
D. Haelew. 4202b	*Laboulbenia insigninoda* nom. prov.	*Pallodes pallidus* (Palisot de Beauvois, 1817)	United States of America	PP620900	PP626226	PP620982		PP601370
D. Haelew. 4203a	*Laboulbenia insigninoda* nom. prov.	*Pallodes pallidus*	United States of America	PP620901	PP626227	PP620983		PP601371
D. Haelew. 4197b	*Laboulbenia mairei*	*Heterocerus fenestratus* (Thunberg, 1784)	Belgium	OR680725	OR680722	OR680745		
D. Haelew. 4573a	*Laboulbenia metableti*	*Syntomus foveatus* (Geoffroy, 1785)	The Netherlands	PP620902	PP626228	PP620984		
D. Haelew. 4334a	*Laboulbenia murmanica*	*Bembidion* sp.	Canada	PP620903	PP626229	PP620985		
D. Haelew. 4193a	*Laboulbenia notiophili*	*Demetrias monostigma* Samouelle, 1819	The Netherlands	PP620904	PP626230	PP620986		
D. Haelew. 4235a	*Laboulbenia notiophili*	*Notiophilus biguttatus* (Fabricius, 1779)	Belgium	PP620905	PP626231	PP620987		PP601372
D. Haelew. 4476a	*Laboulbenia notiophili*	*Paradromius linearis*	Belgium	PP620906	PP626232	PP620967		
D. Haelew. 4728a	*Laboulbenia notiophili*	*Paradromius linearis*	The Netherlands	PP620907	PP626233	PP620988		
D. Haelew. 4083a	*Laboulbenia ophoni*	*Ophonus rufibarbis* (Fabricius, 1792)	Belgium	PP620908	PP626234	PP620989		
D. Haelew. 4714a	*Laboulbenia ophoni*	*Ophonus rufibarbis*	Belgium	PP620909		PP620990		
D. Haelew. 3062a	*Laboulbenia pedicellata*	*Bembidion striatum* (Fabricius, 1792)	Latvia	PP620910	PP626235	PP620991		
D. Haelew. 3230b	*Laboulbenia pedicellata*	*Dyschirius angustatus* (Ahrens, 1830)	Latvia	PP620911		PP620992		
D. Haelew. 4383a	*Laboulbenia pedicellata*	*Bembidion guttula* (Fabricius, 1792)	The Netherlands	PP620912	PP626236	PP620993		
D. Haelew. 4392a	*Laboulbenia pedicellata*	*Bembidion guttula*	The Netherlands	PP620913	PP626237	PP620994		
D. Haelew. 4173a	*Laboulbenia pedicellata*	*Bembidion minimum* (Fabricius, 1792)	Belgium	PP620914	PP626238	PP620995		PP601373
D. Haelew. 4173b	*Laboulbenia pedicellata*	*Bembidion minimum*	Belgium	PP620915	PP626239	PP620996		PP601374
D. Haelew. 3061a	*Laboulbenia pedicellata*	*Bembidion tenellum* Erichson, 1837	Latvia	PP620916		PP620997		
D. Haelew. 4195c	*Laboulbenia perplexa*	*Galerita championi* Bates, 1884	Honduras	PP620917		PP620998		
D. Haelew. 1009a*	*Laboulbenia pheropsophi*	*Pheropsophus* sp.	Sierra Leone			PP620999		
D. Haelew. 1009b*	*Laboulbenia pheropsophi*	*Pheropsophus* sp.	Sierra Leone			OR680760		
D. Haelew. 4581a	*Laboulbenia pseudomasei*	*Pterostichus niger* (Schaller, 1783)	Belgium	PP620918		PP621000		
D. Haelew. 4678b	*Laboulbenia pterostichi*	*Pterostichus* cf. *coracinus* (Newman, 1838)	United States of America	PP620919	PP626240	PP621001		
D. Haelew. 4772b	*Laboulbenia rougetii*	*Brachinus explodens* Duftschmid, 1812	Belgium	PP620920	PP626241	PP621002		
D. Haelew. 4128a	*Laboulbenia slackensis*	*Pogonus chalceus* (Marsham, 1802)	Belgium	PP620921	PP626242	PP621003		
D. Haelew. 4131a (ADK6288)	*Laboulbenia slackensis*	*Pogonus chalceus*	Belgium	OR680737	OR680743	OR680758		
D. Haelew. 4155a	*Laboulbenia slackensis*	*Pogonus chalceus*	Belgium	PP620922	PP626243	PP621004		
D. Haelew. 4190a	*Laboulbenia* sp.	*Agonum extensicolle*	United States of America	PP620923				
D. Haelew. 1113d*	*Laboulbenia* sp.	*Alagoasa* sp.	Panama			PP621005		
D. Haelew. 3756a	*Laboulbenia* sp.	*Amara aenea* (De Geer, 1774)	Belgium	PP620924	PP626244	PP621006		
D. Haelew. 4256a	*Laboulbenia* sp.	*Amara apricaria* (Paykull, 1790)	The Netherlands	PP620925	PP626255	PP621007		
D. Haelew. 4090c	*Laboulbenia* sp.	*Bembidion atrocaeruleum* (Stephens, 1828)	Belgium	PP620926				
D. Haelew. 967a*	*Laboulbenia* sp.	Chrysomelidae	Panama	PP620927				
D. Haelew. 4715a	*Laboulbenia* sp.	*Parophonus maculicornis* (Duftschmid, 1812)	Belgium	PP620928	PP626245	PP621008		
D. Haelew. 4645b	*Laboulbenia* sp.	*Platynus tenuicollis* (LeConte, 1848)	United States of America	PP620929	PP626246	PP621009		
D. Haelew. 4645c	*Laboulbenia* sp.	*Platynus tenuicollis*	United States of America	PP620930				
D. Haelew. 4199b	*Laboulbenia spissa* nom. prov.	*Cyparium concolor* (Fabricius, 1801)	United States of America	PP620931				
D. Haelew. 4199c	*Laboulbenia spissa* nom. prov.	*Cyparium concolor*	United States of America	OR680730		OR680751		
D. Haelew. 4199d	*Laboulbenia spissa* nom. prov.	*Cyparium concolor*	United States of America	OR680731		OR680750		
D. Haelew. 4199e	*Laboulbenia spissa* nom. prov.	*Cyparium concolor*	United States of America	PP620932		PP621010		
D. Haelew. 4057a	*Laboulbenia stilicicola*	*Rugilus* sp.	Belgium	PP620933	PP626247	PP621011		
D. Haelew. 4057c	*Laboulbenia stilicicola*	*Rugilus* sp.	Belgium	PP620934	PP626248	PP621012		
D. Haelew. 3962c	*Laboulbenia temperei*	*Chaetocnema* cf. *hortensis* (Geoffroy, 1785)	United Kingdom	PP620935		PP621013		
D. Haelew. 3982d	*Laboulbenia thaxteri*	*Asaphidion flavipes* (Linnaeus, 1760)	Belgium	PP620936	PP626249	PP621014		PP601375
D. Haelew. 4062a	*Laboulbenia thaxteri*	*Asaphidion flavipes*	Belgium	PP620937				
D. Haelew. 4064a	*Laboulbenia thaxteri*	*Asaphidion flavipes*	Belgium	PP620938		PP621015		
D. Haelew. 3777a	*Laboulbenia vulgaris*	*Bembidion dentellum* (Thunberg, 1787)	The Netherlands	PP620939	PP626250	PP621016		
D. Haelew. 4059a	*Laboulbenia vulgaris*	*Bembidion dentellum*	Belgium	PP620940				
D. Haelew. 3068a	*Laboulbenia vulgaris*	*Bembidion lampros* (Herbst, 1784)	Belgium	PP620941				
D. Haelew. 4711a	*Laboulbenia vulgaris*	*Bembidion lampros*	Belgium	PP620942		PP621017		
D. Haelew. 4375a	*Laboulbenia vulgaris*	*Bembidion tetracolum* Say, 1823	Belgium	PP620943	PP626251	PP621018		
D. Haelew. 3776a	*Laboulbenia vulgaris*	*Bembidion tetracolum*	The Netherlands	PP620944				
D. Haelew. 3776b	*Laboulbenia vulgaris*	*Bembidion tetracolum*	The Netherlands	PP620945	PP626252	PP621019		
D. Haelew. 4231b	*Laboulbenia vulgaris*	*Bembidion tetracolum*	The Netherlands	PP620946	PP626253	PP621020		
D. Haelew. 3069a	*Laboulbenia vulgaris*	*Bembidion tibiale* (Duftschmid, 1812)	Belgium	PP620947		PP621021		
D. Haelew. 3774a	*Laboulbenia vulgaris*	*Bembidion tibiale*	The Netherlands	OR680733	OR680740	OR680753		
D. Haelew. 3775a	*Laboulbenia vulgaris*	*Bembidion tibiale*	The Netherlands	PP620949	PP626254	PP621022		

The ‘CombSIL’ PCR protocol (acronym for Combination of nrSSU, ITS, nrLSU) is a combination of the PCR protocols for nrSSU, ITS, and nrLSU. These separate protocols all use the same annealing temperatures, and similar timings for each step. By combining them into one protocol, we can use the same PCR machine to amplify these different regions at the same time. When using the old protocol to amplify *TEF1* (Table 2: *TEF1*), if bands were visible, there were often multiple bands. When increasing the annealing temperature, clear single bands were observed on the gel after staining (Table 2: *TEF1* New).

## Discussion

### Comparison of preservation methods

The preservation method had a significant effect on the probability to successfully extract DNA of Laboulbeniales. Thalli stored in a doubtful way (in 70% ethanol or dried and pinned for a prolonged time) had a significantly lower probability of successful DNA extraction than thalli collected and stored in > 95% ethanol. This was already reported in a few studies on *Laboulbeniales* (Weir and Blackwell 2001; Haelewaters et al. 2015, 2019a). Anecdotally, we observed lower extraction success for thalli that were stored for more than 4–6 months (between collection and DNA extraction) in 70% ethanol. A more detailed and standardized experiment should be conducted to study the effect of different preservations methods on DNA extractions success of *Laboulbeniales*, including short-term versus longer-term preservation on 70% ethanol, 96% ethanol (expensive molecular grade and cheap denatured bio-ethanol), RNAlater, CTAB, isopropanol, and on -20 °C.

The REPLI-g Kit utilizes a Multiple Displacement Amplification (MDA) to amplify DNA during the WGA step, which involves random hexamer primers and phi29 polymerase (Long et al. 2020). MDA makes use of primers that randomly link to multiple sites of the DNA template and thus no target-specific primers are needed. Disadvantages of MDA include incomplete coverage and over-representation of certain fragments in the resulting DNA extract by chance (e.g., multi-copy regions). If DNA is fragmented, amplicons may be short of even absent, leading to incomplete amplification of the whole-genomic DNA. Subsequent PCR of a specific region is likely to fail due to fragmentation in the primer annealing sites or in the target amplicon. DNA fragmentation is expected in thalli when hosts were stored dried or in 70% ethanol for a prolonged time (> 4 months), as observed in other organisms (Bruns et al. 1990; Kates et al. 2021). For successful molecular work on *Laboulbeniales*, it is essential to perform the first steps of DNA extraction protocols immediately after collection, or to directly transfer of host specimens to > 95% ethanol, according to our current knowledge.

While this result was expected, it is crucial to emphasize the value of collections made by collaborators and entomologists as a critical resource for *Laboulbeniales* research (Haelewaters et al. 2015, 2021). Unfortunately, these collections are often inadequately preserved in 70% ethanol or dried and pinned for extended periods, as it is standard procedure in entomological research. For instance, in a 2022 collection of carabid beetles, only 12 out of 41 DNA extractions were successful, likely due to preservation in 70% ethanol for 6–12 months, even though the specimens were collected and processed within the past year (Fig. 2B). For other organisms it is known that their DNA will degrade in 70% ethanol after three months (Flournoy et al. 1996) to one year (Nagy 2010).

For the purpose of molecular work involving Sanger sequencing, we encourage collectors and collaborators to store their collections in > 95% ethanol, with a single ethanol refreshment (1 to 4 weeks after collection) to maintain the required concentration, as ethanol can extract water from host specimens and *Laboulbeniales* (Nagy 2010; Marquina et al. 2021). Taking proactive measures and collaborating with potential partners can maximize the utility of future collections for various entomological and mycological research purposes. Laboulbeniologists should seize these opportunities to investigate diverse aspects of these understudied insect-associated fungi, encompassing alpha taxonomy, ecology, evolution, and molecular research.

However, preliminary data suggest that preservation on > 95% ethanol and long-term preservation of DNA extracts (post-MDA) at -20 °C may not not useful for other applications such as whole genome sequencing (D. Haelewaters and C.A. Quandt, unpubl. data). Additionally, preservation in ethanol destroys the biofilms present on the thallus surfaces of *Laboulbeniales*, impeding the study of these unknown communities (Lubbers et al. 2022).

### Comparison of extraction protocols and DNA concentrations

We had a high probability to successfully extract DNA of *Laboulbenia* species (0.824 < π < 0.873) if material was preserved correctly, compared to the 0% and 20% reported previously (Haelewaters et al. 2015). There was no difference in the probability of obtaining a successful DNA extraction between the different protocols (halved and normal) and with or without the addition of BSA. There was one exception: this probability is significantly lower for the ‘*No BSA and halved*’ group compared to the and ‘*BSA and halved*’ group, which is likely the result of the small sample size of the former group and an artifact of the number of samples used that were stored in a doubtful way. First of all, this means that we can use the halved protocol to make DNA extractions. This way, twice the number of extractions can be made with the same kit. A single DNA extraction using the REPLI-g Single Cell Kit costs between 24 and 30 euros. When halving the amount of resources per extraction, the costs per extraction are also halved. Yet, the extracts using the halved protocol are still more expensive than other widely used extraction protocols (Lickfeldt et al. 2002; Romanelli et al. 2014; Lahuf et al. 2019). The search for time- and cost-efficient and reliable DNA extraction methods for *Laboulbeniales* continues.

Secondly, the addition of BSA did not result in a significantly higher probability to obtain a successful extraction. There are multiple questions and considerations that arise with this result. It is harder to extract DNA of fungi that contain high concentrations of melanin(-like) molecules. These molecules confer rigidity and protection to the cells (Butler and Day 1998). In *Laboulbeniales*, melanin has been suggested to be the reason for the low DNA extraction successes (Haelewaters et al. 2015; Sundberg et al. 2018a). Thalli of *Laboulbenia* contain variable amounts of melanin(-like) molecules in their cell walls, but their concentration is unknown. Giambernardi et al. (1998) found that the addition of more than 0.5 µg melanin to a 25-µl assay results in the inhibition of *Taq* polymerase. Assuming that a thallus of an average species of *Laboulbenia* is a cylinder with a length of 350 µm, a diameter of 60 µm, and a density between 0.1 and 1.3 g/cm^3^ (Bakken and Olsen 1983), results in an estimated thallus weight between 0.099 and 1.29 µg. This would mean that the amount of melanin needed to inhibit PCR (0.5 µg/25 µl) is either higher than the lowest estimated weight of a thallus (0.099 µg) or more than a third of the highest estimated weight (1.29 µg), which seems unlikely. In other words, we think that the melanin content in the thallus cells is insufficient to hinder WGA or PCR. Consequently, the addition of BSA would have little impact on the probability of successful DNA extraction. It is possible, though, that other molecules such as phenols or proteins, potentially in combination with melanin(-like) molecules, could impede WGA or PCR.

The difference in DNA concentration between the halved and normal REPLI-g protocol was expected, as reagents used to amplify the genome are halved, only half of the amount of DNA can be amplified. There is still some variation in the DNA concentration within each group, which is probably because the amount of the REPLI-g mix pipetted into the PCR tubes slightly differed between samples due to small pipetting errors. The number of thalli used did not have a significant effect on the obtained DNA concentrations. This means that using one thallus is sufficient to make a successful DNA extraction using the REPLI-g Single Cell Kit. However, some species are very small, and using multiple thalli ensures that at least a few pieces of thalli will end up in the PBS solution when transferring them into the PCR tube.

We measured the A260/A280 and A260/A230 ratios to assess the purity of the extracted and amplified DNA. The measurements of all except one undiluted DNA extractions were not within the ideal absorbance ratio range for ‘pure’ DNA of 1.7–2.0 (for A260/A280) and 1.9–2.2 (for A260/A230). This is not surprising, as BSA was added to all these extractions, and other contaminants (other proteins, melanin, and phenols) are potentially present. Most diluted DNA extractions were, based on these absorbance ratios, more purified. As the DNA is diluted, the amount of contaminants is also diluted. There is an interest to sequence whole genomes of *Laboulbeniomycetes* to study the evolution of fungal genomes, population genetics, speciation patterns, parasite–host interactions, and the loss of hyphae for which good quality and ‘pure’ DNA extractions are needed. Haelewaters et al. (2020) sequenced the first *Laboulbeniomycetes* genome, of *Herpomyces periplanetae*. To further purify DNA extractions of *Laboulbeniales*, PCR inhibitor removal kits like the OneStep PCR Inhibitor Removal Kit (Zymo Research, Irvine, CA) (Hu et al. 2015; Vicente et al. 2019; Lubbers 2021) and SPRI bead cleaning (Beckman Coulter, Brea, CA) (B. Young and W. Van Caenegem, unpubl. data) can be used. This approach seems promising for future applications.

### Evaluation of primer pairs, PCR protocols, and sequence success

Generating sequences of *Laboulbenia* species has generally been regarded as difficult (Haelewaters et al. 2015; Sundberg et al. 2018a). Here we generated the highest number of sequences of *Laboulbenia* in a single study, using the REPLI-g Single Cell Kit.

The NSL1/NSL2 primer pair works very well for species in the genus *Laboulbenia*. This pair specifically amplifies *Laboulbeniomycetes* DNA, thus it can also be used even when DNA extractions are contaminated (Haelewaters et al. 2015). While the nrSSU marker is generally not useful for species delimitation as it is a very conserved region (but see Paloi et al. 2022 for a discussion on group I introns), it can be used to distinguish higher taxa (genera, family, order).

The general fungal primers for ITS do not sufficiently amplify the whole ITS region of *Laboulbenia* species. Although we did have some success using ITS1f/ITS4 and ITS5/ITS4, there is a considerable difference with the amplification success of nrSSU and nrLSU region. At the end of the trial, we used the ITS3/ITS4 pair to amplify the ITS2 region. Surprisingly, this generated positive results for most taxa. It is likely that there is a primer mismatch at the primer annealing sites of ITS1f and ITS5, and, for some species, of ITS4 (e.g., *Laboulbenia cristata*, *L. galeritae*, and *L. gyrinicola*), as previously suggested (Liu et al. 2020). Bellemain et al. (2010) reported a bias for *Ascomycota* in eDNA studies when using ITS2, ITS3, and ITS4, while the forward primers ITS1, ITS1f, and ITS5 show a bias towards *Basidiomycota*. This might explain why we successfully amplified the ITS2 region, but not the ITS1 nor the whole ITS region for most species.

For the nrLSU region, the primer pair NL1/NL4 works well. The amplicon is around 300 base pairs shorter than the one amplified by LR0R/LR5. However, LR0R/LR5 did not work for most taxa of *Laboulbenia* and if it worked, multiple faint bands were often observed on the gel after visualization. Similar observations were made with extractions of *Gloeandromyces* and *Hesperomyces* (W. Van Caenegem and D. Haelewaters, unpubl. data). Multiple bands on the gel might indicate that the annealing temperature used (50 °C; Table 2) was suboptimal for this primer pair (Rychlik et al. 1990). Increasing the annealing temperature might overcome this problem, but there is also likely a primer mismatch in most species of *Laboulbenia* for one or both primers.

Although NL1/NL4 works very well for *Laboulbenia* species, it also amplifies the DNA of contaminants. Species of two genera (*Malassezia* and *Priceomyces*) were observed multiple times. *Malassezia* species (*Ustilaginomycotina*, *Malasseziales*) are basidiomycetous yeasts that live on the skin of humans and other vertebrates (Theelen et al. 2018). These species are probably present in our DNA extractions due to the many handlings needed during the extraction process (Fig. 1). *Priceomyces vitoshaensis* (*Saccharomycotina*, *Saccharomycetales*) is an ascomycetous yeast described from the carabid beetle *Pterostichus melas* (Crous et al. 2016). There is not much known about the ecology of this species, but other species of *Priceomyces* are found in eDNA studies of soil, on beetles, in beetle guts, and in insect frass (Kurtzman 2011; Groenewald et al. 2018; Kudo et al. 2019). The micromanipulation needed to remove thalli from the host, may increase contamination.

We could not successfully amplify the three other markers (*MCM7*, *TEF1*, and mtSSU). Some sequences of *MCM7* and *TEF1* were generated for *Laboulbenia* species, but we decided to not explore these markers and their primers further to save time, money, and resources. Recently, *MCM7* was introduced as a secondary marker in *Hesperomyces* (Haelewaters et al. 2022; Van Caenegem et al. 2023b) and *TEF1* is easily amplified for *Gloeandromyces* species (Liu et al. 2020; Van Caenegem et al. 2023a). Amplifying these regions is more challenging for species of *Laboulbenia* compared to our experiences with *Gloeandromyces*, *Hesperomyces*, and *Nycteromyces* (W. Van Caenegem and D. Haelewaters, unpubl. data). The first amplification trial of mtSSU yielded no sequences and we therefore made no further use of the MS1/MS2 primer pair. Sundberg et al. (2018b, 2018a) generated mtSSU sequences of *Laboulbeniales* and reported this region to be “the easiest to amplify and as well as sequence.” However, they used the primer pair mrSSU1/mrSSU3R and they mostly sequenced *Coreomyces* species, which in part may explain the difference in amplification success.

To overcome these challenges of contaminants, primer mismatches, and low amplification success of several loci, we propose to design *Laboulbeniales*- and more specifically *Laboulbenia*-specific primers, especially for the ITS and nrLSU regions, similar to the *Laboulbeniomycetes*-specific nrSSU primers (NSL1/NSL2) (Haelewaters et al. 2015) and the *Hesperomyces*-specific ITS primers (ITSHespL/ITSHespR) (Haelewaters et al. 2018a). These developments will not only help to overcome these aforementioned challenges within the genus *Laboulbenia*, but they will also contribute to the study of *Laboulbeniales* in general, as most species remain unsequenced, resulting in the underestimation of the true diversity of these understudied microfungi as well as a poor understanding of evolutionary relationships due to under-sampling.

## Conclusions

Using the REPLI-g Single Cell Kit, we made 111 successful DNA extractions from *Laboulbenia* species. The preservation method had a major effect on the success of DNA extraction; specimens stored for extended periods in 70% ethanol or dried and pinned were found unsuitable for molecular work. For future research, we encourage entomologists, collaborators, and collectors to preserve infected host specimens in > 95% ethanol. Our findings revealed no significant differences in DNA extraction protocols, indicating that utilizing half the recommended amount is sufficient to successfully extract DNA—saving costs. Further, the addition of BSA did not significantly impact the probability of obtaining successful DNA extractions, suggesting that the melanin content in *Laboulbenia* species is negligible. We generated 104 nrSSU, 64 ITS, 92 nrLSU, 1 *MCM7*, and 16 *TEF1* sequences. The limited success in amplifying protein-coding genes can likely be attributed to primer mismatches. Furthermore, a disparity in amplification success between ITS and the nrSSU and nrLSU regions was observed, likely stemming from primer mismatches. It is imperative to develop *Laboulbeniales*-specific ITS and nrLSU primers to tackle contaminations and improve amplification efficiency. These new insights do not only apply to the genus *Laboulbenia*; the vast majority *Laboulbeniales* species remain unsequenced. The study of *Laboulbeniales* will benefit from the molecular developments reported here. And while we keep critically evaluating and improving our methods, we hope that other research groups will be inspired to start molecular work with these peculiar microfungi.

## Data Availability

The datasets supporting the conclusions of this article are available in the GitHub repository, https://github.com/dannyhaelewaters/teamlaboul/tree/main/molecular_laboulbenia_paper. Newly generated sequences were submitted to the National Center for Biotechnology Information (NCBI) GenBank database (https://www.ncbi.nlm.nih.gov/genbank/), under the accession numbers indicated in Table 6.

## Abbreviations

BSA: Bovine serum albumin
CI: Confidence interval
eDNA: Environmental DNA
EMM: Estimated Marginal Means
ITS: Internal transcribed spacer
MDA: Multiple Displacement Amplification
mtSSU: Mitochondrial small subunit
NCBI: National Center for Biotechnology Information
nrLSU: Nuclear large subunit ribosomal RNA
nrSSU: Nuclear small subunit ribosomal RNA
PBS: Phosphate-buffered saline
RPM: Revolutions per minute
sc: Single cell
WGA: Whole-genome amplification
